# Identification and annotation of breed-specific single nucleotide polymorphisms in *Bos taurus* genomes

**DOI:** 10.1371/journal.pone.0198419

**Published:** 2018-06-01

**Authors:** Bartosz Czech, Magdalena Frąszczak, Magda Mielczarek, Joanna Szyda

**Affiliations:** 1 Biostatistics Group, Department of Genetics, Wroclaw University of Environmental and Life Sciences, Wroclaw, Poland; 2 Institute of Animal Breeding, Balice, Poland; University of Illinois, UNITED STATES

## Abstract

In *Bos taurus* the universality of the reference genome is biased towards genetic variation represented by only two related individuals representing the same Hereford breed. Therefore, results of genetic analyses based on this reference may not be reliable. The 1000 Bull Genomes resource allows for identification of breed-specific polymorphisms and for the construction of breed-specific reference genomes. Whole-genome sequences or 936 bulls allowed us to construct seven breed specific reference genomes of *Bos taurus* for Angus, Brown Swiss, Fleckvieh, Hereford, Jersey, Limousin and Simmental. In order to identify breed-specific variants all detected SNPs were filtered within-breed to satisfy criteria of the number of missing genotypes not higher than 7% and the alternative allele frequency equal to unity. The highest number of breed-specific SNPs was identified for Jersey (130,070) and the lowest—for the Simmental breed (197). Such breed-specific polymorphisms were annotated to coding regions overlapping with 78 genes in Angus, 140 in Brown Swiss, 132 in Fleckvieh, 100 in Hereford, 643 in Jersey, 10 in Limousin and no genes in Simmental. For most of the breeds, the majority of breed-specific variants from coding regions was synonymous. However, most of Fleckvieh-specific and Hereford-specific polymorphisms were missense mutations. Since the identified variants are characteristic for the analysed breeds, they form the basis of phenotypic differences observed between them, which result from different breeding programmes. Breed-specific reference genomes can enhance the accuracy of SNP driven inferences such as Genome-wide Association Studies or SNP genotype imputation.

## Introduction

A reference genome is a DNA database, assembled as a representative of species' nucleotide sequence. Typically, it is based on genomic sequences from many individuals and, as a result, it is universal for the species. Nevertheless, we must be aware of the fact that it represents genetic diversity averaged over the polymorphisms represented in DNA of donor(s) genomes. It should also be borne in mind that most species are represented by distinctive breeds with specific phenotypic and thus also genotypic characteristics. By relating individual DNA sequence variation to the one universal template, we encounter a danger to miss population-specific variation, since all polymorphism detections are “biased” towards the sequence structure represented by the reference genome. That is why breed-specific reference genomes are essential for providing more accurate genomic inferences, which account for DNA sequence variation among particular breeds [1, 2]. Such diversity occurs due to breed formation history, differences in selection pressure as well as migration barriers. In *Bos taurus* the universality of the UMD3.1 reference genome is highly biased towards genetic variation represented by only two related individuals representing the same Hereford breed–a bull “L1 Domino 99375” and his daughter “L1 Dominette 01449” [3]. Therefore, results of genetic analysis of cattle breeds based on the UMD3.1 reference may not be reliable [4].

Decreasing costs of obtaining individual whole genome sequences (WGS) enabled generation of large data sets consisting of DNA WGS of many individuals representing various breeds or populations. Such an initiative has been inaugurated as the 1000 Genomes Project for humans, which now harbours WGS for 3,900 individuals from 30 populations [5]. In 2010, the 1000 Genome initiative has also been developed for cattle [6, 7], for which the current database (run 6 from 2017) comprises WGS of 2,333 individuals from over 60 breeds.

This resource allows for identification of breed-specific polymorphisms and furthermore for construction of breed-specific reference genomes. This procedure was a major goal of our study and was conducted for Angus, Brown Swiss, Fleckvieh, Hereford, Jersey, Limousin and Simmental breeds represented by a large number of individual whole genome DNA sequences. However, since the analysed breeds differ in many aspects such as meatiness, milkiness, fertility and so on, genomic differences between breeds were translated into the functional genomic features by functional annotation of breed-specific SNPs.

## Materials and methods

### Single nucleotide polymorphisms

**Fig 1:**
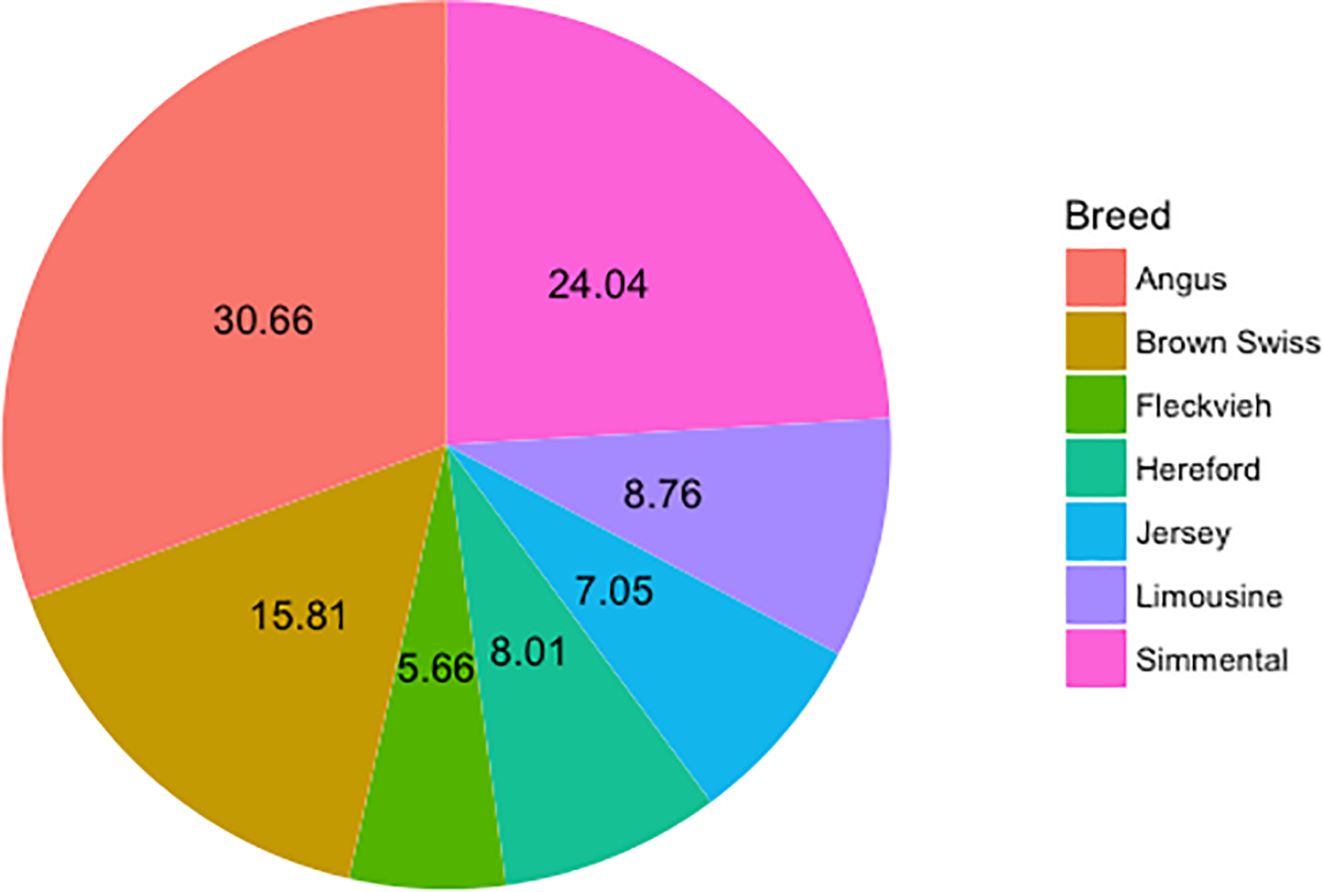
The percentages of individuals representing each breed.

The material comprised whole genome DNA sequences of 936 bulls representing Angus (287 bulls), Brown Swiss (148), Fleckvieh (53), Hereford (75), Jersey (66), Limousin (82) and Simmental (225) breeds available within the frame of the 1000 Bull Genome project [6]. The seven breeds were selected out of the 72 breeds with WGS available, based on two criteria comprising the relatively large number of sequenced bulls and diverse production types. Note, that despite the largest number of available WGSs, the Holstein-Friesian breed was intentionally excluded from the analysis. Since Holstein and Fleckvieh share a common population history prior and during the domestication process [8], the Holstein-Friesian breed, which represents the most highly selected dairy breed worldwide, was excluded from the comparisons, as we believe selection has had a strong influence on the available genetic variation. Whereas for all the other considered breeds selection seems to be more moderate. The distribution of bulls across breeds was not uniform with nearly one-third (30.66%) of sequenced animals, representing the Angus breed, followed by the Simmental breed (24.04%) and Brown Swiss breed (15.81%). Other breeds were represented by a much lower fraction of bulls of less than 10% each (Fig 1).

Table 1 presents descriptive statistics of genome average coverage of the data. The highest mean of genome average coverage was available for the Angus breed (16.71 ±12.18) and the lowest mean was observed for the Fleckvieh breed (7.61 ± 3.24). The bioinformatic pipeline underlying variant detection implemented by the 1000 Bull Genomes Consortium was as follows. The bioinformatic pipeline underlying variant detection implemented by the 1000 Bull Genomes Consortium was as follows. The alignment to this reference genome was performed using the BWA-MEM software [9]. The resulting alignment files (BAM) were further processed by realignment around indels to minimize mismatches across all reads. For this purpose GATK tools RealignerTargerCreator and IndelRealigner were applied [10]. The detection of SNPs was carried out with SAMtools [11] simultaneously for all bulls representing a given breed. Filters were then implemented in the VCF file using PyVCF.

**Table 1:** Descriptive statistics of distribution of genome average coverage.

Breed	Minimum	Maximum	Mean (SD)
Angus	2.57	72.46	16.71 (±12.18)
Brown Swiss	5.46	35.71	11.78 (±3.9)
Fleckvieh	4.35	25.35	7.61 (± 3.24)
Hereford	4.35	64.54	14.67 (± 8.38)
Jersey	2.65	28.37	11.07 (± 4.27)
Limousin	3.85	37.41	10.80 (± 6.56)
Simmental	2.72	39.30	11.70 (± 5,77)

### Defining breed-specific SNPs

In our analysis, aiming to identify breed-specific SNPs, the first step was to remove variants with two or more alternative alleles. The first filtering criterion was selection of a minimum number of alternative alleles observed on a forward and a reverse strand reads (threshold used was 1—removes variants never observed on forward and reverse reads). Then variants with overall quality below 20 and mapping quality below 30 were removed. The next criterion for filtering was selecting the minimum and maximum of read depth (respectively min = 10 and maximum = median of read depth + 3 standard deviation of read depth). Then two variants assigned the same base pair position were removed. Subsequently InDels, which revealed another InDel closer than 10 base pairs, SNPs, which revealed another SNP closer than 3 base pairs and SNPs closer than 5 base pairs to InDels were removed.

### Genomic and functional annotation

In order to identify breed-specific variants all SNPs were filtered within-breed to satisfy criteria of the number of missing genotypes not higher than 7% and the alternative allele frequency equal to unity using he VCF Tools software [12]. Those filtered polymorphisms that were characteristic to only one breed (and absent in the other breeds) were defined as breed-specific. Those SNPs were genomically annotated using the Variant Effect Predictor [13] and functionally annotated to Gene Ontology and KEGG pathways using the KOBAS software [14]. The last step was to prepare FASTA files representing breed-specific reference genomes obtained by replacing the selected nucleotides in the UMD3.1 template.

## Results and discussion

### Quantitative characteristic of SNPs

The total number of identified SNPs and the number of SNPs remaining after the filtration comprising the number of missing genotypes not higher than 7% and the alternative allele frequency equal to unity was summarized in Table 2. The highest total number of SNPs was observed in the Simmental breed (61,824,209) and the lowest number in Fleckvieh (61,780,912), with the highest percentage of SNPs after the filtration remaining for Jersey (0.494%) and the lowest percentage for Simmental (0.098%).

**Table 2:** The total number of SNPs and the number of SNPs after the filtration.

Breed	Total number of SNPs	Number of SNPs after the filtration	% of SNPs after the filtration
Angus	61,823,312	114,744	0.186
Brown Swiss	61,814,873	187,576	0.303
Fleckvieh	61,780,912	189,238	0.306
Hereford	61,813,028	73,772	0.119
Jersey	61,797,641	305,477	0.494
Limousin	61,820,287	61,623	0.100
Simmental	61,824,209	60,582	0.098

**Fig 2:**
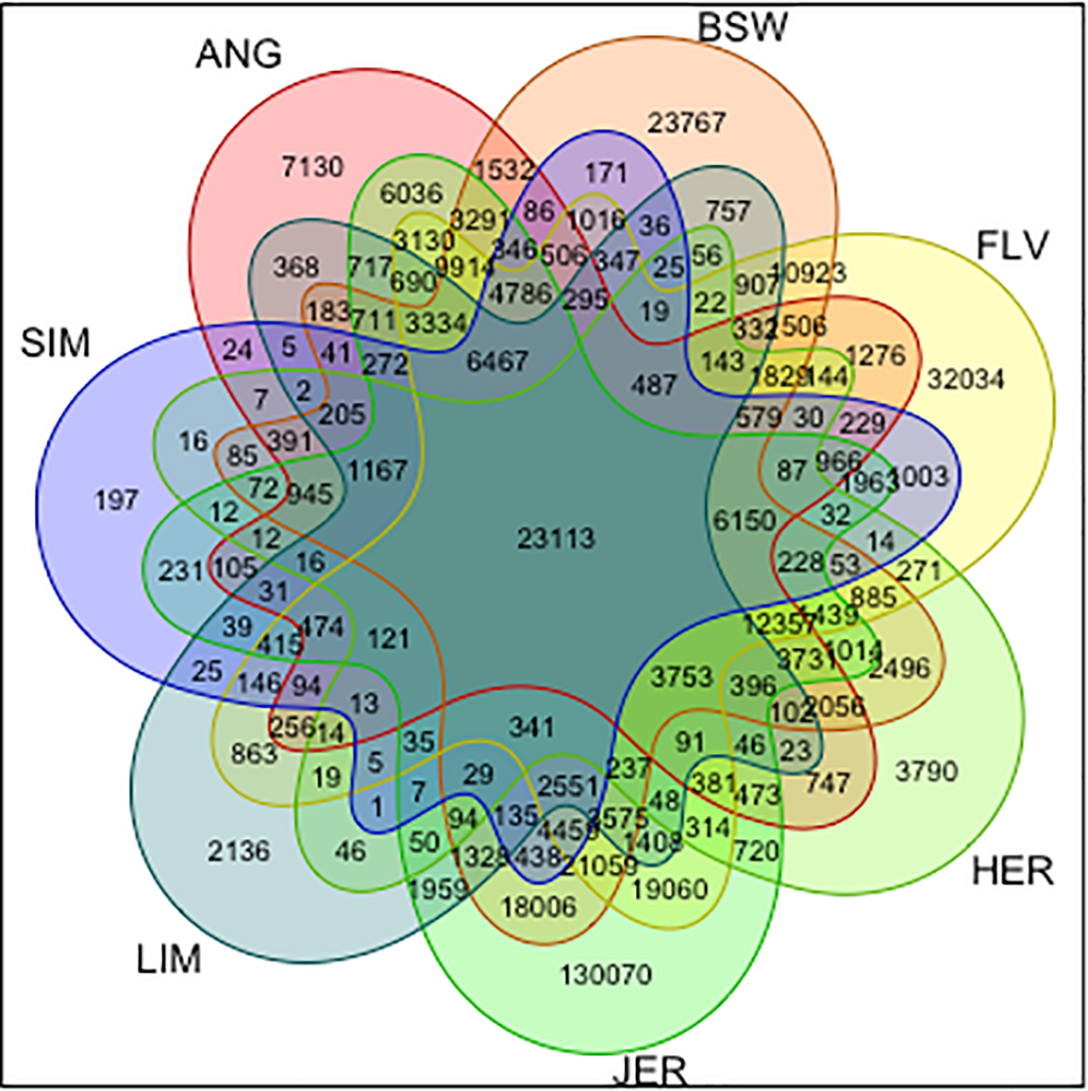
A Venn diagram of breed-specific SNPs. Each number represents the count of breed-specific SNPs, which are common between sets of breeds, defined by colours. ANG–Angus, BSW–Brown Swiss, FLV–Fleckvieh, HER–Hereford, JER–Jersey, LIM–Limousin, SIM–Simmental.

The post-filtration variants were visualised using the Venn diagram (Fig 2) showing the numbers of SNPs common between all possible breed combinations. 23,113 of filtered SNPs were common to each breed, which indicated a polymorphism characteristic to Domino lineage, but monomorphic in the other breeds. Surprisingly, as many as 3,790 SNPs were alternative homozygous in Hereford, again indicating that the polymorphisms are rather Domino-Dominette specific then a characteristic of *Bos taurs* as a species. Still a considerable number of variants were specific for only one breed. The highest number of breed-specific SNPs was identified for Jersey (130,070) and the lowest—for the Simmental breed (197). Furthermore, considering pairwise breed comparisons, the highest numbers of SNPs specific only for a combination of two breeds was identified for Jersey-Fleckvieh (19,060) and for Jersey-Brown Swiss (18,006) combinations, the latter may have been expected provided phenotypic similarities between both breeds as well as a similar milk production performance. The lowest numbers of breed-combination specific SNPs were always attributable to constellations involving the Simmental breed, i.e. Simmental-Hereford (16 SNPs), Simmental-Angus (24 SNPs), Simmental-Brown Swiss (171 SNPs) and Simmental-Jersey (231 SNPs). This remains in agreement with results presented by [15] who showed the largest phylogenetic distance between Simmental and the other breeds considered in our study. The only exception was a relatively high number of SNPs characteristic to the Simmental-Fleckvieh combination–which reflects a common origin of both breeds. They are even regarded as the same breed, with only traditional differentiation into Fleckvieh in Germany and Simmental in other countries (see e.g. a common breeding value evaluation for Fleckvieh and Simmental conducted by the Interbull Center www.interbull.org).

### Genomic and functional annotation of breed-specific SNPs

**Fig 3:**
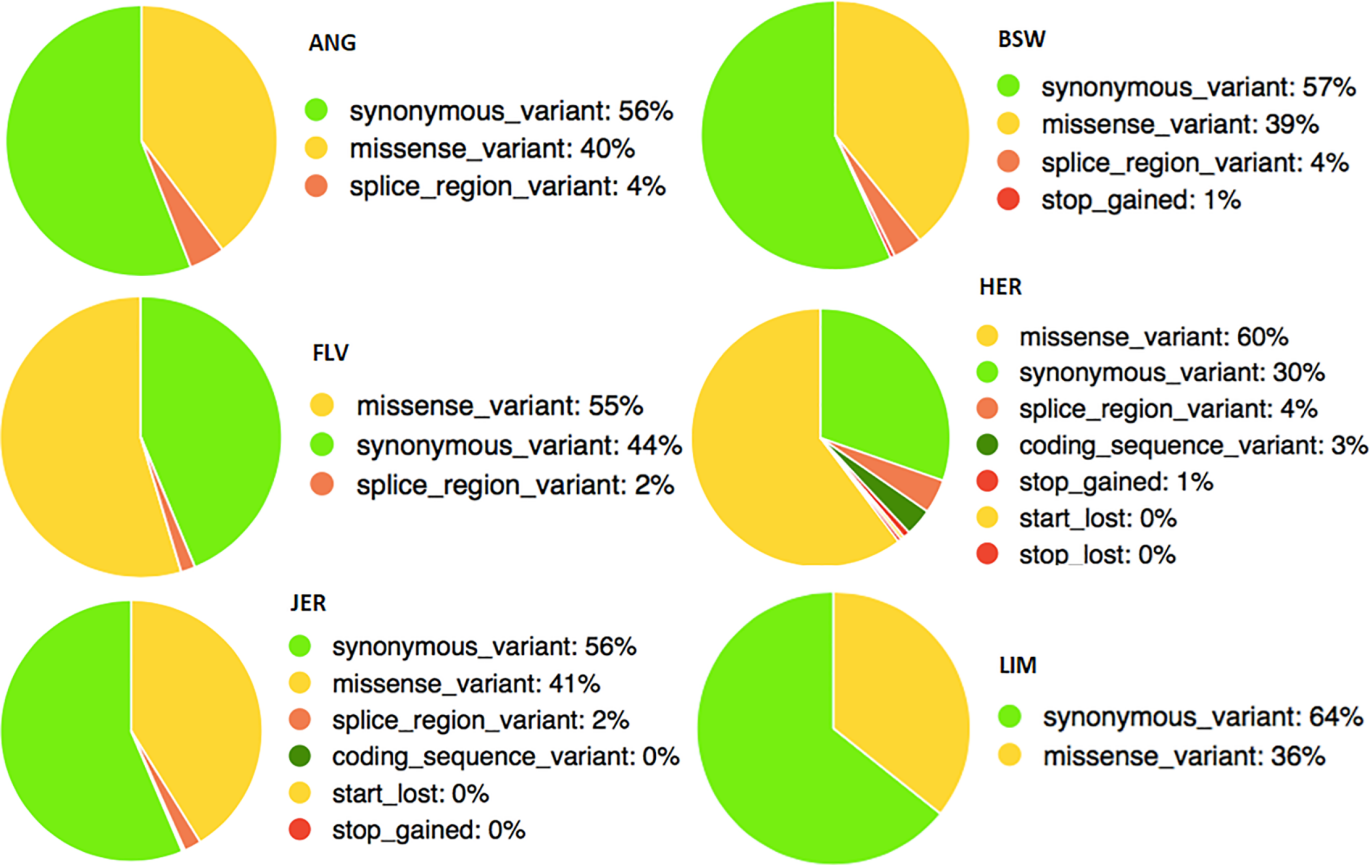
Functional annotation of breed-specific SNPs to coding regions. ANG–Angus, BSW–Brown Swiss, FLV–Fleckvieh, HER–Hereford, JER–Jersey, LIM–Limousin.

SNPs identified as breed-specific were genomically annotated to the UMD3.1 reference. Polymorphisms located in coding regions overlapped with 78 genes in Angus, 140 in Brown Swiss, 132 in Fleckvieh, 100 in Hereford, 643 in Jersey and 10 in Limousin. Note, that due to a low number of only 197 Simmental-specific SNPs, no polymorphisms located in coding regions were identified for this breed. Since such polymorphisms, potentially contribute to differences between breeds, we considered their functional consequences expressed by Sequence Ontology terms (Fig 3).

As it might have been expected, in most of the breeds over the half of breed-specific variants located within coding regions was synonymous. However, the sets of Fleckvieh-specific and Hereford-specific polymorphisms were exceptions, since for those two breeds there were missense mutations, which composed the most of SNPs (FLV– 55%, i.e. 100 SNPs and HER– 60%, i.e. 141 SNPs). Also for the remaining breeds, the number of missense point mutations was considerable ranging from 41% (485 SNPs) in Jersey to 36% (5 SNPs) in Limousin. Besides missense mutations, albeit less numerous, breed-specific SNPs with other high impact consequences were identified. These comprised SNPs located in splice regions, exonic SNPs resulting in a premature termination or prolongation of a translation process, as well as SNPs which alter the translation start codons. Further on, based on KEGG pathways, we checked whether whole metabolic processes representing interplay of several genes and their products, were specifically altered in the analysed breeds. Our results showed that genes harbouring breed-specific mutations in coding regions do not significantly represent any particular pathway.

Phenotypic annotation of breed-specific SNPs to the OMIA data base (omia.org) has been done using the BioMart software [16]. Genes harbouring breed-specific SNPs and related to OMIA phenotypes have been identified for Angus, Brown Swiss, Jersey and Limousin. Two of the genes represented the same gene family (solute carrier), which encodes membrane transport proteins: SLC39A4 on BTA14 related to Acrodermatitis enteropathica, contained an Angus-specific SNP and SLC4A2 on BTA4 related to Osteopetrosis, contained a Brown Swiss-specific SNP. Interestingly, the link between a mutation causing Acrodermatitis enteropathica and SLC39A4 was reported for the Angus breed [17]. Osteopetrosis in calves caused by a 2,750 long deletion in SLC4A2 and was reported for multiple breeds, but not Brown Swiss. For Brown Swiss we also identified a breed-specific SNP within the LAMA3 gene on BTA24. A mutation in this gene was reported to cause an Epidermolysis bullosa, junctionalis disease in Belgian Blue cattle [18]. Jersey-specific mutations were located within four genes harbouring disease mutations. The GON4L gene on BTA3 with a single bp deletion causing dwarfism in Fleckvieh [19], contained three Jersey-specific SNPs, the APOB gene on BTA11 contains a deletion, which causes cholesterol deficiency in German Holstein breed [20], the TG gene on BTA14 is responsible for familial goitre [21], and the COL7A1 gene on BTA22 with a mutation resulting in the an epidermolysis bullosa disorder [22, 23]. In addition, three Limousin-specific SNPs were located within the PFAS gene on BTA19, which harbours a mutation responsible for an abortion (due to haplotype MH1) identified by [24] in Montbéliarde cattle. Since the analysed data set represents phenotypically healthy individuals–none of the abovementioned disease coding mutations is identical with breed-specific SNPs. However, the three Jersey-specific SNPs within the GON4L gene were located in the same exon as the disease causing SNP.

Next, it was checked which genes harbouring breed-specific SNPs overlapped with QTL found in the QTLdb (www.animalgenome.org/cgi-bin/QTLdb/BT/index). Genes with mutations characteristic for the Angus breed overlapped with QTL related to iron content in mussels, which is supported by the earlier study [25] demonstrating a different (lower) iron content of iron in Angus and Charolais than in Simmental and Limousin. For the Brown Swiss breed we linked genes with breed-specific SNPs to many QTL related to milk characteristics: milk solids percentage, milk overall proteins percentage, milk alpha-casein percentage, milk alpha-lactalbumin percentage, milk kappa-casein percentage, milk beta-casein percentage, milk lactose content and yield; udder health expressed by somatic cell score; growth traits: body weight, and longissimus muscle area; and gastrointestinal nematode burden. This collection of traits controlled by genes with Brown Swiss-specific variation reflects a production character of the breed with the balance between beef and dairy character with milk composition suitable for chees production. For the remaining breeds, has not been identified QTL associated genes.

## Conclusions

In cattle phenotypic differences between breeds are much more pronounced than in humans. They are enhanced by strong artificial selection for divergent production goals, like dairy or beef. Such phenotypic differences are driven by underlying changes in the genome structure, which emphasises an importance of breed-specific genomic inferences, for which breed-specific reference genomes are the prerequisite. Breed-specific reference genomes can enhance the accuracy of SNP based inferences such as Genome-wide Association Studies or SNP genotype imputation. In the future, when specific reference genomes will be available for many breeds, they can form a basis for describing interbreed volatility and dynamics of changes in the genome of *Bos taurus* caused by breeding programmes [26].

## References

[1] Cho YS, Kim H, Kim H-M, Jho S, Jun J, Lee YJ, et al An ethnically relevant consensus Korean reference genome is a step towards personal reference genomes. Nat Commun. 2016; 7:13637 doi: 10.1038/ncomms13637 27882922 10.1038/ncomms13637 PMC5123046

[2] Maretty L, Jensen JM, Petersen B, Sibbesen JA, Liu S, Villesen P, et al Sequencing and de novo assembly of 150 genomes from Denmark as a population reference. Nature. 2017; 548:87–91. doi: 10.1038/nature23264 28746312 10.1038/nature23264

[3] Zimin AV, Delcher AL, Florea L, Kelley DR, Schatz MC, Puiu D, et al A whole-genome assembly of the domestic cow, *Bos taurus*. Genome Biol. 2009; 10:R42 doi: 10.1186/gb-2009-10-4-r42 19393038 10.1186/gb-2009-10-4-r42 PMC2688933

[4] Eck SH, Benet-Pagès A, Flisikowski K, Meitinger T, Fries R, Strom TM. Whole genome sequencing of a single *Bos taurus* animal for single nucleotide polymorphism discovery. Genome Biol. 2009; 10:R82 doi: 10.1186/gb-2009-10-8-r82 19660108 10.1186/gb-2009-10-8-r82 PMC2745763

[5] 1000 Genomes project. A global reference for human genetic variation. Nature. 2015; 526:68–74. doi: 10.1038/nature15393 26432245 10.1038/nature15393 PMC4750478

[6] Hayes BJ, Fries R, Lund MS, Boichard DA, Stothard P, Veerkamp RF, et al. 1000 Bull Genomes Consortium Project. Plant and Animal Genome XX Conference, San Diego, CA, USA, 14–18 January 2012

[7] Daetwyler HD, Capitan A, Pausch H, Stothard P, van Binsbergen R, Brøndum RF, et al Whole-genome sequencing of 234 bulls facilitates mapping of monogenic and complex traits in cattle. Nat Genet. 2014; 46:858–65. doi: 10.1038/ng.3034 25017103 10.1038/ng.3034

[8] Boitard S, Rodríguez W, Jay F, Mona S, Austerlitz F. Inferring Population Size History from Large Samples of Genome-Wide Molecular Data—An Approximate Bayesian Computation Approach. PLoS Genet. 2016; 12:e1005877 doi: 10.1371/journal.pgen.1005877 26943927 10.1371/journal.pgen.1005877 PMC4778914

[9] Li H, Durbin R. Fast and accurate short read alignment with Burrows–Wheeler transform. Bioinformatics. 2009; 25:1754–1760. doi: 10.1093/bioinformatics/btp324 19451168 10.1093/bioinformatics/btp324 PMC2705234

[10] McKenna A, Hanna M, Banks E, Sivachenko A, Cibulskis K, Kernytsky A, et al The Genome Analysis Toolkit: a MapReduce framework for analyzing next-generation DNA sequencing data. Genome Res. 2010; 20:1297–1303. doi: 10.1101/gr.107524.110 20644199 10.1101/gr.107524.110 PMC2928508

[11] Li H., Handsaker B., Wysoker A., Fennell T., Ruan J., Homer N. The Sequence alignment/map (SAM) format and SAMtools. Bioinformatics. 2009; 25:2078–2079 doi: 10.1093/bioinformatics/btp352 19505943 10.1093/bioinformatics/btp352 PMC2723002

[12] Danecek P, Auton A, Abecasis G, Albers CA, Banks E, DePristo MA The variant call format and VCFtools. Bioinformatics. 2010; 27:2156–2158. doi: 10.1093/bioinformatics/btr330 21653522 10.1093/bioinformatics/btr330 PMC3137218

[13] McLaren W, Gil L, Hunt SE, Riat HS, Ritchie GRS, Thormann A. The Ensembl Variant Effect Predictor. Genome Biol. 2016; 17:122 doi: 10.1186/s13059-016-0974-4 27268795 10.1186/s13059-016-0974-4 PMC4893825

[14] Xie C, Mao X, Huang J, Ding Y, Wu J, Dong S, et al KOBAS 2.0: a web server for annotation and identification of enriched pathways and diseases. Nucleic Acids Res. 2011; 39:W316–322. doi: 10.1093/nar/gkr483 21715386 10.1093/nar/gkr483 PMC3125809

[15] Decker JE, McKay SD, Rolf MM, Kim J, Molina Alcalá A, Sonstegard TS, et al Worldwide patterns of ancestry, divergence, and admixture in domesticated cattle. PLoS Genet. 2014; 10:e1004254 doi: 10.1371/journal.pgen.1004254 24675901 10.1371/journal.pgen.1004254 PMC3967955

[16] Kinsella RJ, Kähäri A, Haider S, Zamora J, Proctor G, Spudich G, et al Ensembl BioMarts: a hub for data retrieval across taxonomic space. Database (Oxford). 2011; bar030. doi: 10.1093/database/bar030 21785142 10.1093/database/bar030 PMC3170168

[17] Tammen I, Cook RW, Gitschier J, Nicholas FW, Raadsma HW. Mapping and identification of the gene causing hereditary zinc deficiency in Angus cattle Proceedings of the 28th International Conference on Animal Genetics in Göttingen, 2002:70.

[18] Sartelet A, Harland C, Tamma N, Karim L, Bayrou C, Li W, et al A stop-gain in the laminin, alpha 3 gene causes recessive junctional epidermolysis bullosa in Belgian Blue cattle. Anim Genet. 2015; 46:566–570. doi: 10.1111/age.12342 26370913 10.1111/age.12342

[19] Schwarzenbacher H, Wurmser C, Flisikowski K, Misurova L, Jung S, Langenmayer MC, et al A frameshift mutation in GON4L is associated with proportionate dwarfism in Fleckvieh cattle. Genet Sel Evol. 2016; 48:25 doi: 10.1186/s12711-016-0207-z 27036302 10.1186/s12711-016-0207-z PMC4818447

[20] Menzi F, Besuchet-Schmutz N, Fragnière M, Hofstetter S, Jagannathan V, Mock T, et al A transposable element insertion in APOB causes cholesterol deficiency in Holstein cattle. Anim Genet. 2016; 47:253–257. doi: 10.1111/age.12410 26763170 10.1111/age.12410 PMC4849205

[21] Ricketts MH, Simons MJ, Parma J, Mercken L, Dong Q, Vassart G. A nonsense mutation causes hereditary goitre in the Afrikander cattle and unmasks alternative splicing of thyroglobulin transcripts. P Natl Acad Sci USA. 1987; 84:3181–3184. 10.1073/pnas.84.10.3181 PMC304832 3472203

[22] Menoud A, Welle M, Tetens J, Lichtner P, Drögemüller C. A COL7A1 Mutation Causes Dystrophic Epidermolysis Bullosa in Rotes Höhenvieh Cattle. PLoS One. 2012; 7:e38823 doi: 10.1371/journal.pone.0038823 22715415 10.1371/journal.pone.0038823 PMC3371016

[23] Pausch H, Ammermüller S, Wurmser C, Hamann H, Tetens J, Drögemüller C, et al A nonsense mutation in the COL7A1 gene causes epidermolysis bullosa in Vorderwald cattle. BMC Genet. 2011; 17:149 doi: 10.1186/s12863-016-0458-2 27905875 10.1186/s12863-016-0458-2 PMC5131490

[24] Fritz S, Capitan A, Djari A, Rodriguez SC, Barbat A, Baur A, et al Detection of Haplotypes Associated with Prenatal Death in Dairy Cattle and Identification of Deleterious Mutations in GART, SHBG and SLC37A2. PLoS ONE. 2016; 8:e65550 doi: 10.1371/journal.pone.0065550 23762392 10.1371/journal.pone.0065550 PMC3676330

[25] Chambaz A, Scheeder MR, Kreuzer M, Dufey PA. Meat quality of Angus, Simmental, Charolais and Limousin steers compared at the same intramuscular fat content. Meat Sci. 2003; 63:491–500 22062519 10.1016/s0309-1740(02)00109-2

[26] The Bovine HapMap Consortium. Genome-wide survey of SNP variation uncovers the genetic structure of cattle breeds. Science. 2009; 324:528–532. doi: 10.1126/science.1167936 19390050 10.1126/science.1167936 PMC2735092

